# Hypothalamic GHR—SIRT1 Axis in Fasting

**DOI:** 10.3390/cells10040891

**Published:** 2021-04-14

**Authors:** Juliana Bezerra Medeiros de Lima, Chidera Ubah, Lucas Kniess Debarba, Iven Ayyar, Olesya Didyuk, Marianna Sadagurski

**Affiliations:** Department of Biological Sciences, Integrative Biosciences Center (IBio), Wayne State University, Detroit, Michigan, MI 48202, USA; lima@wayne.edu (J.B.M.d.L.); gi9232@wayne.edu (C.U.); lucaskniess@wayne.edu (L.K.D.); iven.ayyar@wayne.edu (I.A.); olesya.didyuk@wayne.edu (O.D.)

**Keywords:** growth hormone axis, growth hormone receptor, Sirtuin 1, hypothalamus, arcuate nucleus

## Abstract

Many aspects of physiological functions are controlled by the hypothalamus, a brain region that connects the neuroendocrine system to whole-body metabolism. Growth hormone (GH) and the GH receptor (GHR) are expressed in hypothalamic regions known to participate in the regulation of feeding and whole-body energy homeostasis. Sirtuin 1 (SIRT1) is the most conserved mamma-lian nicotinamide adenine dinucleotide (NAD^+^)-dependent protein deacetylase that plays a key role in controlling life span and sensing nutrient availability in the hypothalamus in response to caloric restriction. However, the interaction between GHR signaling and SIRT1 in the hypothal-amus is not established. In the arcuate nucleus (ARC) of the hypothalamus, the anorexigenic proopiomelanocortin (POMC)-expressing neurons and the orexigenic agouti-related protein (AgRP)-expressing neurons are the major regulators of feeding and energy expenditure. We show that in the ARC, the majority of GHR-expressing neurons also express SIRT1 and respond to fasting by upregulating SIRT1 expression. Accordingly, hypothalamic upregulation of SIRT1 in response to fasting is blunted in animals with GHR deletion in the AgRP neurons (AgRP^EYFPΔGHR^). Our data thus reveal a novel interaction between GH and SIRT1 in responses to fasting.

## 1. Introduction

The majority of physiological functions including control of metabolism, reproduction, circadian rhythm, and energy homeostasis are regulated by the central nervous system (CNS), specifically by the hypothalamus [1]. The hypothalamic neural network within the arcuate nucleus (ARC), ventromedial (VMH), dorsomedial (DMH), paraventricular nucleus (PVN), and lateral hypothalamus (LH) controls nutrient homeostasis and energy balance. In the ARC, an opposed interaction between agouti-related peptide (AgRP)-expressing neurons and proopiomelanocortin (POMC)-expressing neurons constitute the central metabolic axis [2,3]. Our previous studies have highlighted the involvement of hypothalamic growth hormone receptor (GHR) signaling in regulating glucose metabolism [4]. A recent study demonstrated the capacity of AgRP GHR knock out male mice to save energy during a 60% food restriction, indicating the role of hypothalamic GHR during caloric restriction (CR) [5].

In mammals, the beneficial effects of CR on the metabolism have been linked to the function of the sirtuin family of NAD^+^-dependent protein deacetylases (SIRT1 in mice) [6]. The overexpression of SIRT1 in the brain promotes energy balance in response to CR [7], particularly in the hypothalamic DMH and LH nuclei [8]. SIRT1 is highly expressed in the ARC [7,9]. However, there have been conflicting reports demonstrating changes in SIRT1 expression triggered by fasting [10]. The selective deletion of SIRT1 in AgRP neurons or pharmacological inhibition with EX-527 decreased food intake, led to decreased lean mass, fat mass, and body weight [11,12]. On the other hand, genetic overexpression of SIRT1 in AgRP neurons suppressed food intake [7,13]. Taken together, these studies illustrate the role of hypothalamic SIRT1 in food intake; however, the complexity of the system is still far from being clear.

Numerous studies indicate potential interaction between SIRT1 and the GH axis. It was suggested that CR triggers a reduction in neuronal SIRT1 activity in hypothalamic neurons by lowering GH signaling [14]. In agreement, the inhibition of SIRT1 reduces neuronal insulin growth factor 1 (IGF-1) signaling by the inhibition of Ras/MEK1/ERK1/2 activity, which was sufficient to protect the neurons against oxidative stress [15]. This effect is in contrast with the activation of SIRT1 by CR in many other peripheral tissues. For example, in the liver, SIRT1 activation reduces IGF-1 serum levels [16], while IGF-binding protein-1 (IGFBP-1) levels are significantly increased in SIRT1 KO mice [17]. On the other hand, while fasting reduces SIRT1 levels in skeletal muscle [18], resveratrol supplementation had no impact on IGF-1 levels or GH signaling in human muscle and fat [19]. Overall, these data indicate that SIRT1 can modulate the GH/IGF-1 axis in a tissue-specific and context-dependent manner.

Alterations in GH/IGF-1 signaling and SIRT1 signaling have effects on aging and age-dependent diseases. The effects of these pathways on life span are variable depending on the animal model, and they are tissue-specific. The mechanism by which these pathways affect life span in mice is far less well defined. In this study, we show that GHR-expressing neurons are co-localized with SIRT1 in the hypothalamic ARC, and we suggest a novel role of SIRT1 within the GH axis in the AgRP^GHR+^ neurons in response to fasting. Future investigation of a possible link between the somatotropic system and SIRT1 function in the hypothalamus is important for the maintenance of whole-body energy homeostasis and possibly for the regulation of life span.

## 2. Materials and Methods

### 2.1. Animals

Adult male and female AgRP^tm1(cre)^(AgRP-Ires-cre, stock 012899), tdTomato mice on ROSA26 background (B6.Cg-Gt(ROSA)26Sort^m14(CAG-tdTomato)Hze^/J, stock 007914) and yellow fluorescent protein (EYFP) mice on ROSA26 background (B6.129 × 1-Gt(ROSA)26Sor^tm1(EYFP)Cos^/J, stock 006148) were purchased from the Jackson Laboratory, and GHR^L/L^ mice were described previously [20]. All mice were provided ad libitum access to a standard chow diet (Purina Lab Diet 5001, St. Louis, MO, USA) and housed in temperature-controlled rooms on a 12-h/12-h light–dark cycle. We used wild-type littermate control male and female mice whenever possible, and if not available we used age-matched controls from the same breeding line.

Lean and fat body mass were assessed by a Bruker Minispec LF 90II NMR-based device. GH levels were determined on fasted mice by the Rat/Mouse Growth Hormone ELISA kit (Millipore, Burlington, MA, USA), and IGF-1 levels were determined by Mouse/Rat IGF-1 ELISA kit (22IG1MSE01, Alpco, Salem, NH, USA). For peripheral GH stimulation (12.5 µg/100 g body weight, GroPep Bioreagents Pty Ltd., Adelaide, Australia), mice were injected intraperitoneal (i.p.) and perfused 1.5 h later for pSTAT5 immunostaining as before [4]. All procedures and experiments were carried out in accordance with the guidelines established by the National Institutes of Health Guide for the Care and Use of Laboratory Animals, and approved by the Wayne State University Institutional Animal Care and Use Committee.

### 2.2. Perfusion and Histology

Mice were anesthetized (IP) with avertin and transcardially perfused with phosphate-buffered saline (PBS) (pH 7.5) followed by 4% paraformaldehyde (PFA). The brains were post-fixed, sank in 30% sucrose, frozen in Optimal cutting temperature compound (OCT) medium, and then sectioned coronally (30 µm) and processed for immunohistochemistry as previously described [21,22]. For immunohistochemistry, free-floating brain sections were washed in PBS, blocked using 3% normal donkey serum (NDS) and 3% Triton X-100 in PBS, and then stained with a primary antibody overnight in blocking buffer. For early gene c-fos immunostaining, sections were pretreated for 20 min in 0.5% NaOH and 0.5% H_2_O_2_ in PBS, followed by immersion in 0.3% glycine for 10 min. Sections were then placed in 0.03% sodium dodecyl sulfate (SDS) for 10 min and placed in 4% normal serum plus 0.4% Triton X-100 plus 1% bovine serum albumin (BSA) for 20 min, and then stained with the following primary antibodies overnight: c-fos (anti-rabbit, 1:500, Santa Cruz, Dallas, TX, USA), pSTAT5 (anti-rabbit, 1:200, #9314, Cell signaling, Danvers, MA, USA) and SIRT1 (anti-mouse, 1:100, ab110304, Abcam, Cambridge, UK). For controlling the specificity of the staining, the immunohistochemical experiments were performed with brain sections in which the primary antibody was omitted and substituted with serum. All floating brain sections were washed with PBS several times and incubated with the anti-rabbit, anti-mouse, or anti-chicken Alexa Fluor 488 and/or 568 (1:200, Invitrogen, Carlsbad, CA, USA) secondary antibody for 2 h. All images were visualized with the Nikon 800 fluorescent microscope using Nikon imaging DS-R12 color-cooled SCMOS, version 5.00.

### 2.3. Stereotaxic Trichostatin A Intracerebroventricular Injection

Trichostatin A (TSA) or vehicle was injected as previously described [23]. Briefly, 8- to 12-week-old AgRP^EYFPΔGHR^ and control female mice were fasted for 18 h before the experiment. One hour before perfusion, the mice were induced to anesthesia in a 5% isoflurane chamber and then placed in a stereotaxic apparatus (model 1900, Kopf Instruments, Tujunga, CA, USA) under 0.8% isoflurane anesthesia. Injections of 0.5 µL (10 µg/µL) were performed using and Hamilton microliter glass syringe (model 7001, Hamilton, Reno, NV, USA) coupled to a micropump (UMP3T-1 UltraMicroPump 3, World Precision Instruments, Sarasota, FL), with a volume injection rate of 250 nL/min into the third ventricle (3V). The 3V coordinates in relation to bregma were as follows: antero-posterior, −1.70 mm; latero-lateral, 0.00 mm; and dorso-ventral, −5.30 mm. One hour after injections the animals were perfused as described previously.

### 2.4. Ac-Forkhead Transcription Factor (FoxO1) Staining

Free-floating brain sections were washed in tri-buffered saline (TBS) and pretreated for 20 min in 0.5% NaOH and 0.5% H_2_O_2_ in TBS, followed by immersion in 0.3% glycine for 10 min. Then they were placed in 0.03% SDS for 10 min and placed in 4% normal serum plus 0.4% Triton X-100 plus 1% BSA for 20 min, and then stained with the primary antibody overnight (rabbit anti-Ac-FoxO1, 1:100; cat. number MBS9600633, MyBioSource, San Diego, CA, USA). On the next day, brain sections were washed 6 times with TBS at room temperature for 8 min followed by incubation with the biotinylated donkey anti-rabbit antibody (1:200; 711-065-152–lot: 136387, Jackson ImmunoResearch, West Grove, PA, USA) for 2 h. Then, the sections were incubated for 1 h in avidin and biotinylated enzyme (ABC) solution (VECTASTAIN Elite ABC horseradish peroxidase Kit, cat. number PK-6100, Vector Laboratories, Burlingame, CA, USA). The color was developed for 55 s using the Metal Enhanced DAB Substrate Kit (cat. number34065–lot: TB260954, Thermo Scientific, Waltham, MA, USA). Then, the sections were washed 3 times and mounted onto slides, and kept at 4 °C until photomicrographs were taken as described previously.

### 2.5. Statistical Analysis

Unless otherwise stated mean values ± SEM are presented in graphics. The unpaired two-tailed Student’s *t*-test was used for comparisons between two groups. Statistical analyses were performed using the GraphPad Prism software. A *p*-value of less than 0.05 was considered statistically significant.

## 3. Results

### 3.1. GHR-Expressing Neurons Co-Localize with SIRT1

**Figure 1:**
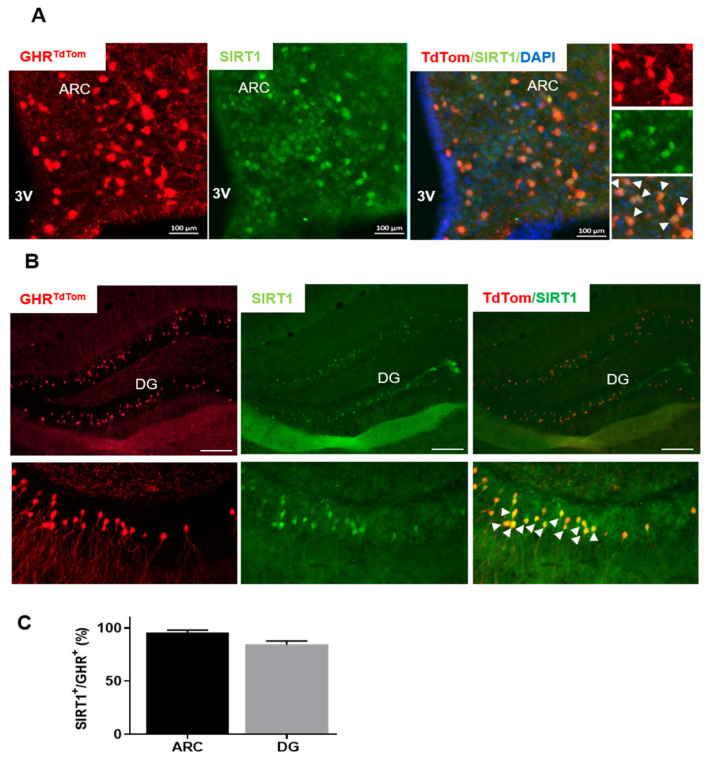
Sirtuin 1 (SIRT1) is co-localized with growth hormone receptor (GHR)^+^ neurons. Representative fluorescence microscopy images of (**A**) the hypothalamus and (**B**) the hippocampus of fasted GHR^tdTom^ mice. Red, tdTomato; green, SIRT1; the merged image shows co-localization between GHR and SIRT1 in the arcuate nucleus (ARC). The magnified images on the right side show the co-localization of tdTomato and SIRT1 fluorescence in the GHR^+^ neurons (arrows). DG, dentate gyrus; ARH or ARC, arcuate nucleus of the hypothalamus. (**C**) The percentage of the GHR^SIRT+^ subpopulation from the total GHR^+^ neurons in the ARC or DG. The data are shown as mean ± SEM. *n* = 4–5. The data were analyzed from both male and female mice. Scale bar: 100 µm. The 3V, third ventricle.

Sirtuin 1 (SIRT1) is highly expressed in the ARC [7,9], and is upregulated upon fasting. Interestingly, several lines of evidence have suggested an interaction between SIRT1 and GHR; however, the data are often contradictory [6,14,16]. To explore the colocalization between GHR-expressing neurons with SIRT1, we utilized our newly generated GHR^tdtom^ mouse model [24,25]. The expression pattern of the tdTomato reporter reveals the presence of GHR-expressing neurons in the hypothalamus, as well as in other previously described brain sites [24,25]. We assessed the co-localization of SIRT1 with GHR-expressing neurons in fasting animals. Interestingly we found that the majority of GHR neurons co-localized with SIRT1 in the hypothalamic ARC and the hippocampus (Figure 1), possibly indicating a role of SIRT1 in the regulation of the somatotropic axis in these neurons.

### 3.2. GHR Signaling in AgRP Neurons Regulates Adaptation to Fasting via SIRT1

**Figure 2:**
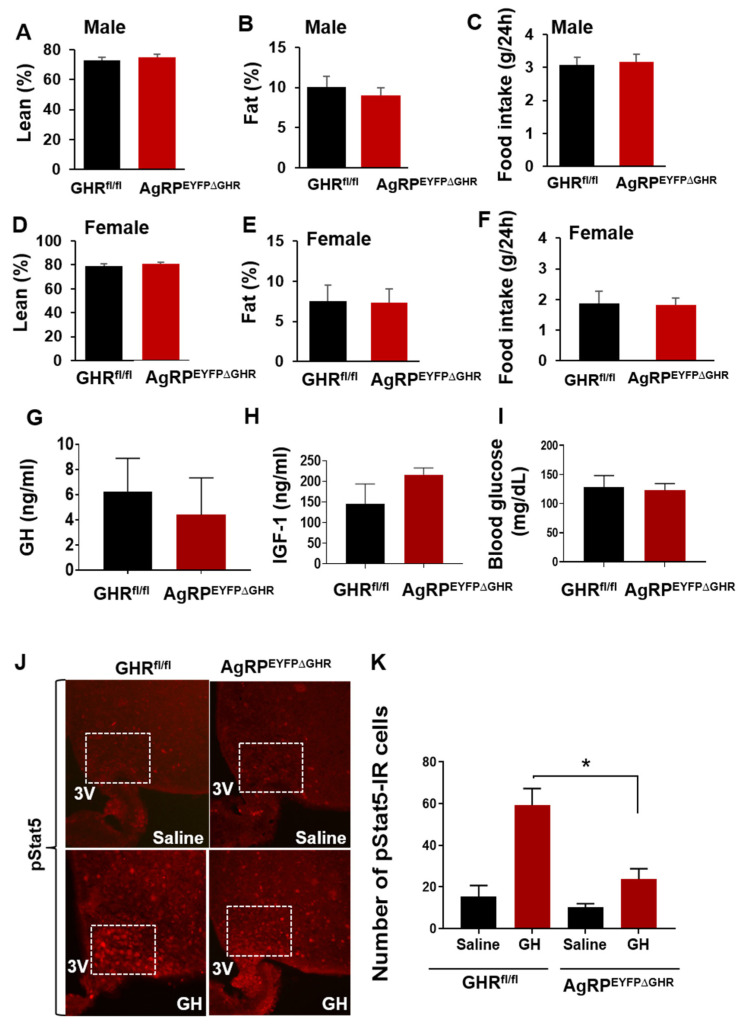
Weight and hormonal levels of agouti-related protein (AgRP)^EYFP∆GHR^ mice. (**A**) Lean mass, (**B**) fat mass, and (**C**) food intake of control and AgRP^EYFP∆GHR^ male mice. (**D**) Lean mass, (**E**) fat mass, and (**F**) food intake of control and AgRP^EYFP∆GHR^ female mice. (**G**) Serum growth hormone (GH), (**H**) serum IGF-1, and (**I**) fasted blood glucose levels of control and AgRP^EYFP∆GHR^ male mice, *n* = 7–8 per group. (**J**) Representative image of STAT5-IR (red) in the ARC of control and AgRP^EYFP∆GHR^ male mice induced by saline or GH (ip). (**K**) Quantification of pSTAT5^+^ cells in the ARC of control and AgRP^EYFP∆GHR^ mice. Mean ± SEM. *, *p* < 0.05. *n* = 4–5. The 3V, third ventricle.

The hypothalamic upregulation of SIRT1 expression following fasting [9] and co-localization with GHR^+^ neurons in the ARC suggests a possible role of SIRT1 in ARC^GHR+^ neurons in metabolic adaptation to fasting. To determine the association between SIRT1 and GHR signaling in AgRP neurons in responses to fasting, we inactivated GHR specifically in AgRP neurons by crossing a well-established AgRP-Ires-cre on cre-inducible ROSA26-EYFP background together with GHR^l/l^ mice (AgRP^EYFPΔGHR^). As evidenced in a recently published study [5], body weight and growth rates of both male and female AgRP^EYFP∆GHR^ mice were comparable to their control littermates (Figure 2A). Body composition revealed similar percentages of fat and lean mass. Accordingly, the food intake of AgRP^EYFP∆GHR^ mice was comparable to their control littermates (Figure 2), suggesting that GHR signaling in AgRP^+^/GHR^+^ neurons does not control normal feeding behavior. Consequently, we assessed serum levels of GH and IGF-1 in control and AgRP^EYFP∆GHR^ mice, but these were not significantly different in either male or female mice (Figure 2G,H). Fasted blood glucose levels were unchanged (Figure 2I). Moreover, while acute intraperitoneal GH injection significantly induced pSTAT5 in the ARC of control mice, the percentage of pSTAT5-IR cells in the ARC of AgRP^EYFP∆GHR^ mice was significantly reduced (Figure 2J,K).

**Figure 3:**
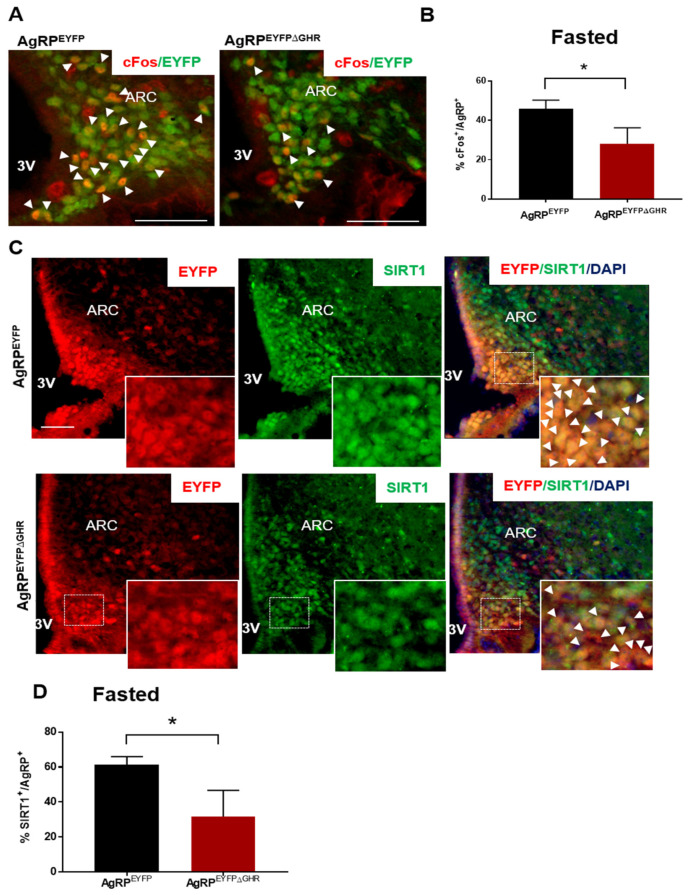
Blunted responses of SIRT1 to fasting in AgRP^EYFPΔGHR^ mice. (**A**) Representative image and (**B**) quantification of 18 h fasting-induced c-fos (red) co-localized with AgRP neurons (red) in AgRP^EYFP∆GHR^ and control mice. (**C**) Representative images of the hypothalamic ARC sections of AgRP^EYFP^ and AgRP^EYFP∆GHR^ female mice fasted for 18 h. Red, AgRP^+^ EYFP neurons; green, SIRT1; the merged images show co-localization of AgRP and SIRT1 in the ARC. Scale bar: 100 µm. The magnified images on the bottom show the co-localization of AgRP and SIRT1 fluorescence. (**D**) The percentage of SIRT1^+^ cells from AgRP^+^ neurons in the ARC. The arrows show co-localization. Data are shown as mean ± SEM. *, *p* < 0.05 (*n* = 3–4).

Unlike control mice that showed significant elevations in c-fos-positive neurons in response to fasting, AgRP^EYFP∆GHR^ mice showed significantly lower c-fos when fasted (Figure 3A,B). This effect was observed in both male and female mice, providing evidence for the role of GHR in AgRP neurons in response to starvation signals, as indicated in a recently published study [5]. Interestingly, the levels of ghrelin or ghrelin-induced c-fos expression in the ARC of these animals was unaltered, suggesting that ghrelin action in AgRP is independent of GHR in ad libitum-fed mice [5]. Immunofluorescence analysis revealed that overnight fasting increased the abundance of SIRT1 within AgRP neurons in control littermates; however, this pattern was significantly diminished in the fasted AgRP^EYFP∆GHR^ mice (Figure 3C). SIRT1 expression was barely detected in both control and AgRP^EYFP∆GHR^ ad lib-fed mice (data not shown).

**Figure 4:**
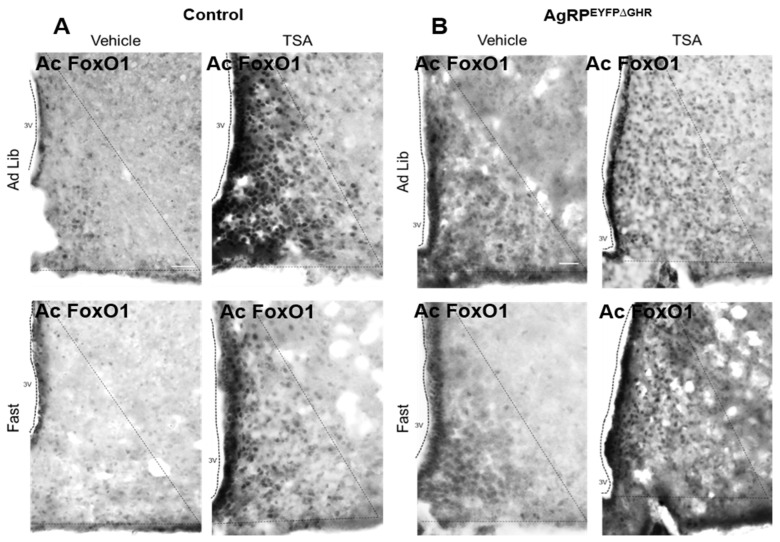
Trichostatin A (TSA) treatment induces forkhead transcription factor (FoxO1) acetylation. Acetylated FoxO1 expression in the ARC of fed and 18-h-fasted female mice. (**A**) The 8–12-week-old female control, and (**B**) AgRP^EYFPΔGHR^ mice were injected with TSA or vehicle into the 3V (Intracerebroventricular: icv). Representative photomicrographs from 3–4 sections per group were pseudo-colored in grayscale. Scale bar: 100 µm. The 3V, third ventricle.

SIRT1 was suggested to regulate fasting by reducing FoxO1 (forkhead transcription factor) acetylation levels [12]. To evaluate the acetylation levels of FoxO1, mice were treated with trichostatin A (TSA), a well-characterized inhibitor of class I and II mammalian histone deacetylase (HDAC) [26]. SIRT1 interacts with HDAC1 and SIRT1-mediated protection is negatively affected by HDAC1 inhibition [26,27,28]. Numerous studies indicate that TSA treatment can increase resistance to oxidative stress, and this effect is similar to EX-527, a specific small-molecule inhibitor of SIRT1. EX-527 and TSA act synergistically on the downstream target of SIRT1, p53 acetylation levels [29]. In the ARC, the acetylation levels of FoxO1 are reduced by fasting; however, TSA treatment markedly induces acetylation of FoxO1 during feed and fasting cycles in control mice (Figure 4). Acetylation levels of FoxO1 were similarly induced in both vehicle and TSA-treated AgRP^EYFP∆GHR^ mice. These results suggest that FoxO1 and its interaction with SIRT1 in the ARC are needed in response to fasting in AgRP^GHR+^ neurons. In the future, imaging of SIRT1 expression−activity using a novel substrate-type radiotracer will be helpful for quantitative molecular analysis of SIRT1 and its interaction with FoxO1 under various feeding conditions [30,31].

## 4. Discussion

Numerous studies demonstrated that SIRT1 is upregulated by fasting [9]. Indeed, we show that the expression of SIRT1 is elevated in AgRP neurons under fasting conditions and impaired in AgRP^EYFP∆GHR^ mice, which suggests that the SIRT1 axis may play an important role in AgRP^+^/GHR^+^ neurons, particularly during fasting. In agreement with these observations, c-fos immunoreactivity is similarly reduced in AgRP^+^/GHR^+^ neurons, providing support for reduced neuronal activation in these mice.

Several studies demonstrated the molecular links between the SIRT1 and GH axis; however, the interaction seems to be tissue- or cell-specific [6,14,16]. On one hand, it was suggested that in the hypothalamus, SIRT1 positively regulates somatotropic signaling, since brain-specific SIRT1 knockout mice are small and have low GH and IGF-1 levels [14]. In neurons, SIRT1 increased insulin/IGF-I signaling by acetylating insulin receptor substrate 2 (IRS-2), followed by Ras/ERK activation and sensitization of primary rat neurons to oxidative stress [15]. On the other hand, in the pituitary SIRT1 pharmacological activation with an antioxidant, resveratrol, suppressed GHRH-induced GH secretion in vivo and in vitro by suppressing cAMP-response element-binding protein (CREB) transcriptional activation [32]. In a study conducted in hepatocytes, it was demonstrated that SIRT1 negatively regulates GH-dependent IGF-I production during the starved condition by deacetylating STAT5 [16]. Based on the data obtained in our study, it is reasonable to hypothesize that SIRT1 in AgRP neurons can act as a hypothalamic gatekeeper that mediates the GHR signaling in adaptive responses to fasting.

CR is known to inhibit the somatotropic GH–IGF-1 signaling pathways. CR associates with marked reductions in serum IGF-1 levels with many other benefits of CR, such as decreased tumor incidence and metabolic abnormalities in rodents [33,34]. Anorexia nervosa (AN) however, is considered starvation or malnutrition. In AN, GH secretion is elevated due to GH resistance, which leads to reduced IGF-I synthesis and release [35,36]; this implies an impairment of the negative IGF-I feedback action on GH secretion [36].

In addition to the tissue- or cell-specific effect of SIRT1, a number of other factors may contribute to the differential regulation of SIRT1. For example, nutrient status, stress response, or subcellular localization of SIRT1 may play a critical role in the regulation of SIRT1 function and potential interaction with the GH axis [37]. This can be especially important given the sensitivity of AgRP and POMC neurons in the ARC to the nutrient status. Indeed, SIRT1 might play distinct roles in these neurons, although the reports regarding the role of hypothalamic SIRT1 in energy balance regulation are mixed. It was demonstrated that the inhibition of hypothalamic SIRT1 increases acetylation of FoxO1, resulting in increased POMC and decreased AgRP expressions [12]. The deletion of SIRT1 from AgRP neurons decreases AgRP neuronal activity, leading to decreased food intake and body weight [11]. On the other hand, SIRT1 overexpression in POMC or AgRP neurons prevented age-associated weight gain; overexpression in POMC neurons stimulated energy expenditure, while SIRT1 overexpression in AgRP neurons suppressed food intake [13]. It is possible, however, that both genetic manipulations resulted in reduced food intake by different mechanisms. Overexpressing SIRT1 in AgRP neurons increased leptin sensitivity, thus suppressing AgRP activity, while the deletion of SIRT1 from AgRP neurons reduced AgRP neuronal excitability. One possible explanation for the differences between these two studies can be related to sexual dimorphism which might include the differences in the dose requirement for SIRT1 [13]. Similarly, only female AgRP-SIRT1 knockout mice demonstrated decreased food intake [11], and only female AgRP-SIRT1-H355Y mice showed increased food intake [38]. Thus, it is possible that AgRP neurons in females are more vulnerable to decreased SIRT1 function. Although the physiological significance and clinical relevance of SIRT1 action in these neurons remain to be clarified, these studies indicate that SIRT1 is critical in mediating normal responses to nutrient availability. Future studies can be facilitated by a recently developed methodology for repetitive non-invasive imaging of SIRT1 expression–activity in the hypothalamus using positron emission tomography (PET) with [18F] BzAHA [31] in knockout or transgenic mice under normal and nutritionally deprived conditions.

There may be at least two mechanisms by which GHR signaling may influence the levels of SIRT1 activity in AgRP neurons. First, by altering systemic metabolism via the activation of AgRP neurons and inducing a CR-like state which indirectly activates SIRT1 in the same and other hypothalamic neurons [7]. The second possible mechanism may involve direct signaling from GHR to SIRT1 via JAK2–STAT5, FoxO1, or other signal transduction pathways [38,39,40]. The acetylation of FoxO1 is reduced in animals during fasting and increased after refeeding [41]. Indeed, we show that the acetylation levels of FoxO1 are reduced by fasting; however, TSA treatment markedly induces acetylation of FoxO1 during feed and fasting cycles in control mice. These findings are in support of studies demonstrating that the inhibition of hypothalamic SIRT1 activity reverses the fasting-induced decrease in FoxO1 acetylation [12,38,42]. Acetylated levels of FoxO1 were induced in AgRP^EYFP∆GHR^ mice regardless of the treatment, consistent with the proposed role of SIRT1, and its substrate FoxO1 [43], in adaptive responses to fasting. The identification of additional molecular mediators in AgRP neurons that are likely to be important within the GH axis and their interaction with SIRT1 will be the next critical step. While further studies will be required, given the importance of SIRT1 in the maintenance of youthful physiology, the investigation of GHR–SIRT1 interaction in the ARC will provide insights into the mechanisms underlying the imbalance of energy homeostasis with aging and possibly into the regulation of longevity in mammals.

## Data Availability

The datasets generated during and/or analyzed during the current study are available from the corresponding author on reasonable request.

## Abbreviations

CNS	central nervous system
GH	growth hormone
GHR	growth hormone receptor
Lepr	leptin receptor
STAT3	signal transducer and activator of transcription 3
STAT5	signal transducer and activator of transcription 5
POMC	proopiomelanocortin
ARC	arcuate nucleus of the hypothalamus
DMH	dorsomedial hypothalamic nucleus
LHA	lateral hypothalamus
PVH	paraventricular hypothalamic nucleus
SIRT1	sirtuin 1
